# Proteomic Analysis of Beef Tenderloin and Flank Assessed Using an Isobaric Tag for Relative and Absolute Quantitation (iTRAQ)

**DOI:** 10.3390/ani10010150

**Published:** 2020-01-16

**Authors:** Zhaomin Lei, Jianping Wu, Deyin Zhang, Ting Liu, Shengguo Zhao, Jianfu Wang, Xiaoxue Zhang

**Affiliations:** 1 College of Animal Science and Technology, Gansu Agricultural University, Lanzhou 730070, China; leizm@gsau.edu.cn (Z.L.); GSAUZDY@163.com (D.Z.); liuting0628@163.com (T.L.); zhaosg@gsau.edu.cn (S.Z.); wangjf@gsau.edu.cn (J.W.); 2 Gansu Academy of Agriculture Sciences, Lanzhou 730070, China; wujp@gsagr.ac.cn

**Keywords:** iTRAQ, beef, proteomics, differential expression, GO analysis, KEGG pathway analysis, tenderloin steak, flank steak

## Abstract

**Simple Summary:**

Amino acid composition is among the important indexes of the nutritional composition of meat nutrients. In this study, we performed a proteomic analysis of tenderloin and flank steaks from Simmental cattle using isobaric tags for a relative and absolute quantification (iTRAQ) approach. Seventeen amino acids were detected in tenderloin and flank steaks, including seven essential amino acids and 10 non-essential amino acids. A comparison of the expression patterns in steaks revealed 128 differentially expressed proteins (DEPs). Furthermore, 27 DEPs (*p* < 0.05) were subjected to Gene Ontology (GO) analysis and Kyoto Encyclopedia of Genes and Genomes (KEGG) pathway analysis.

**Abstract:**

Herein, we performed a proteomic analysis of tenderloin and flank steaks from Simmental cattle using the isobaric tags for a relative and absolute quantification (iTRAQ) approach. We identified 17 amino acids in both steaks, and Gly, Cys, Ile, Lys, and Pro differed most in abundance between the steak types (*p* < 0.05). A comparison of the expression patterns in steaks revealed 128 differentially expressed proteins (DEPs), of which 44 were up-regulated and 84 were down-regulated. Furthermore, 27 DEPs (*p* < 0.05) were subjected to gene ontology (GO) analysis, and many were found to be related to oxidation-reduction, metabolism, hydrogen ion transmembrane transport, transport, the tricarboxylic acid (TCA) cycle, mitochondrial electron transport, and the conversion of nicotinamide adenine dinucleotide (NADH) to ubiquinone. Kyoto Encyclopedia of Genes and Genomes (KEGG) pathway analysis also implicated these DEPs in various signalling pathways, including oxidative phosphorylation, cardiac muscle contraction, the TCA cycle, biosynthesis, and the metabolism. These findings provide a new insight into key proteins involved in the determination of amino acid composition in beef.

## 1. Introduction

Meat quality is crucial in beef cattle production, and mainly focuses on the evaluation of tenderness, juiciness, and flavour [1,2]. Proteomic approaches have been applied to meat science to gain a better understanding of the molecular mechanisms underpinning meat quality [3,4]. It is widely accepted that meat quality is primarily affected by the type and number of myofibers [5]. In mammals, skeletal muscle is a large, metabolically active tissue characterised by high levels of energy metabolism and protein turnover. Proteins are the major constituents of muscle tissue and are responsible for the regulation of metabolic routes involved in the conversion of muscle to meat [6,7]. Protein quality is largely determined by the composition of present amino acids. Thus, a systematic analysis of changes in protein and amino acid abundance may provide a good understanding of meat quality traits for bovine muscle.

Isobaric tagging for relative and absolute quantification (iTRAQ) is a recently developed method for the quantitative analysis of proteomes that can identify proteins and provide more accurate quantitative measurements of protein abundance than traditional two-dimensional electrophoresis analysis [8]. The iTRAQ method has been widely applied to investigate the proteomes of cattle [9], chicken [10], and pig [11]. Previous proteomics studies using iTRAQ have focused on muscle development [11], embryo development [12], meat tenderness [10], and lipid deposition [13] in livestock and poultry. However, differences in protein and amino acid abundance in different types of bovine muscle remain poorly understood.

In the present study, we compared the amino acid content of bovine tenderloin and flank steaks, and identified differentially expressed proteins (DEPs) using advanced iTRAQ analysis. Subsequent bioinformatics analyses, including gene ontology (GO) functional annotation, hierarchical clustering, and Kyoto Encyclopedia of Genes and Genomes (KEGG) pathway assessment, were performed to analyse DEPs between steak types. The findings expand our understanding of the molecular mechanisms underlying bovine meat quality.

## 2. Materials and Methods

### 2.1. Ethics Statement

All procedures involving the use of live animals in experiments were conducted in accordance with the Biological Studies Animal Care and Use Committee (Gansu Province, China). All sampling of animals was approved by the Institutional Animal Care and Use Committee of the Gansu Agricultural University under permit NO. 2012-2-159.

### 2.2. Animals and Sample Collection

Three male Simmental cattle were obtained from Sanyangjinyuannongmu Co. Ltd., Gansu, China. Individuals were randomly selected from weaned calves and reared in a standardised feeding, housing, and management environment for 210 days. The same diet was fed to all cattle, and the total mixed ration (TMR) consisted of a mixed concentrate and corn silage. A seven-phase feeding system was employed, and the nutrition level of each stage was in accordance with the Feeding Standards for beef cattle National Research Council (NRC, USA). Feed and water were provided ad libitum throughout the experiment. Individuals were fasted for 24 h, then water-fasted for 8 h before sacrifice. Cattle were sacrificed by venepuncture for the sampling of the tenderloin and flank steaks. Samples were collected from the same part of the left half of the carcass, immediately snap-frozen in liquid nitrogen, and stored at −80 °C for later use.

### 2.3. Determination of Amino Acids

The amino acid content of the tenderloin and flank steaks was determined using an automatic amino acid analyser (Beijing BangFei Bioscience Co., Ltd., Beijing, China). Briefly, frozen muscle tissue was homogenised with a homogeniser, 30 mg was placed in a hydrolysis tube, 10 mL of 6 M HCl and four drops of freshly distilled phenol were added, the sample was placed in a freezer for 3 min, vacuum-sealed, and hydrolysis proceeded at 110 °C for 24 h. Finally, the concentration was determined using 0.02 M HCl solution.

### 2.4. Extraction of Proteins

Frozen muscle tissue (~50 mg) was homogenised in 0.4 mL of urea buffer (8 M urea, 100 mM TRIS-HCl, 10 mM dithiothreitol [DTT], 1× protease inhibitor, pH 8.0). Homogenates were shaken five times (2 s each time), placed on ice for 20 min, and centrifuged for 30 min at 10,000 g to remove any insoluble components. Protein concentrations were measured using the bicinchroninic acid (BCA) protein quantitation method, and proteins were stored at −80 °C until analysis.

### 2.5. Protein Digestion and iTRAQ Labelling

Each protein (200 μg) was mixed with 1 M DTT (5 μL) for 1 h at 37 °C, 20 μL 1 M indole-3-acetic acid (IAA) was added, and samples were incubated for 1 h at room temperature in the dark. After reaction, 100 μL UA (8 M urea in 100 mM TRIS-HCl, pH 8.5) was added to each sample, samples were centrifuged twice, and proteins were dissolved and centrifuged three times in 100 μL 0.5 M triethylammonium bicarbonate (TEAB). Samples were digested with trypsin (protein:trypsin ratio = 20:1) and incubated at 37 °C for over 12 h. Each peptide pool (~100 μg) was labelled with iTRAQ reagent (flank steaks = 113–115; tenderloin steaks = 117–119) according to the manufacturer’s protocol accompanying the Reagent-8plex Multiplex Kit (AB Sciex, Beijing, China). After labelling, samples were dried and stored at −80 °C for later analysis.

### 2.6. Strong Cation Exchange (SCX) Separation and Liquid Chromatography-Tandem Mass Spectrometry (LC-MS/MS) Analyses

A high-performance liquid chromatography (HPLC) system was used for SCX chromatography. Dry, labelled peptides were reconstituted with 100 μL buffer A (20 mM NH4HCO3 in 3% acetonitrile, pH 10), centrifuged for 20 min at 14,000× *g*, and the supernatant was loaded onto an Ultremex SCX column. Peptides were collected at a flow rate of 0.7 mL/min with buffer B (20 mM NH4HCO3, pH 10) using a gradient of 0–5% for 5 min, 5–35% for 25 min, 35–95% for 7 min, and 95–5% for 8 min. Forty fractions were collected every 1.5 min. These fractions were vacuum dried and re-dissolved in 0.5% formic acid (Sigma) and pooled as six samples. The six SCX fractions were injected using an autosampler and separated by an UltiMate 3000 High-performance liquid chromatography (HPLC) system. Samples were desalted and concentrated on a C18 trap column (3 μm particle size, 0.10 × 20 mm, Beijing BangFei Bioscience Co., Ltd., Beijing, China), then fractionated on a C18 column (1.9 μm, 0.15 × 120mm, BangFei) at a flow rate of 0.6 μL/min. Solvent A was 0.1% formic acid and solvent B was 0.1% formic acid and 80% acetonitrile (can). Elution was carried out with a linear gradient of solvent B from 0–9% for 8 min, 9–14% for 16 min, 14–30% for 36 min, 30–40% for 15 min, 40–95% for 10 min, and 95–100% for 5 min. Peptides were sprayed through a NanoDrop 2000C (Thermo Scientific, Shanghai, China) into a Q-Exactive High-field (HF) mass spectrometer (Thermo Scientific, Shanghai, China).

### 2.7. qRT-PCR

Quantitative real-time reverse transcription PCR (qRT-PCR) was used to detect the expression of genes corresponding to the differentially expressed proteins. Total RNA was extracted from 12 samples using a TRIzol reagent (Invitrogen, Waltham, MA, USA), reverse transcription was performed using a reverse transcriptase kit (Takara, Dalian, China). Real-time PCR was conducted using a SYBR green assay (Takara Biotechnology) on a Roche LightCycler 480 (Roche Applied Science, Mannheim, Germany). The final reaction mixture was 20 µL per sample, which contained 6.4 µL of H_2_O, 0.8 µL of each primer, 2 µL of cDNA, and 10 µL of 2 × SYBR Green PCR Master Mixture (Takara Biotechnology). The reaction protocol was as follows: initial denaturation at 95 °C for 30 s, and 40 cycles of 5s at 95 °C and 30 s at 60 °C. The housekeeping gene *ACTB* (encoding beta actin) was used as an endogenous reference. The primer sequences are listed in the Supplementary Material Table S1. The expression of genes corresponding to the differentially expressed proteins were calculated using the ∆∆Ct method.

### 2.8. Western Blot

Total proteins were extracted from 12 samples based on materials and methods 2.4. Protein samples were separated by 12% sodium dodecylsulfate polyacrylamide gel electrophoresis (SDS-PAGE) and were then transferred onto polyvinylidene difluoride (PVDF) blotting membranes (Beyotime, Shanghai, China). The membranes were blocked with phosphate buffered saline tween-20 (PBST) containing 5% non-fat dry milk for 2 h at room temperature and were then incubated with either a human anti-CSRP3 polyclonal antibody (1:500; Abcam, Shanghai, China), human anti-MYH2 (1:500; Affinity, Shanghai, China) or an anti-beta-actin polyclonal antibody (1:1000; Affinity, Beijing, China) at 4 °C overnight. After being washed with PBST, the membranes were incubated with goat anti-rabbit IgG antibody (1:5000; Bioss, Beijing, China) for 2 h at 37 °C. After being washed with PBST, the membrane was exposed to autoradiography film in an X-ray room, and eventually band intensities were quantified using AlphaEaseFC software (Protein Simple, Santa Clara, CA, USA).

### 2.9. Data Analysis

A statistical analysis of amino acids was performed using Microsoft Excel and IBM SPSS17.0 for Windows Software (SPSS, Chicago, IL, USA). Differences were analysed using independent sample *t*-tests and a one-way analysis of variance (ANOVA), and *p* < 0.05 was considered to indicate statistical significance. Raw data for MS analysis were processed by Mascot2.1 and Proteome Discoverer1.4 (Thermo Scientific, Beijing, China). Raw data were submitted to the Mascot sever by Proteome Discoverer (Beijing BangFei Bioscience Co., Ltd., Beijing, China). Proteins were identified by searching against the uni_bos_taurus_160426.fasta database (Beijing BangFei Bioscience Co., Ltd., Beijing, China) with trypsin as the enzyme and a maximum of two missed cleavages allowed, ± 15 ppm as the peptide mass tolerance, 20 mmu as the fragment mass tolerance, and peptide False discovery rate (FDR) ≤ 0.01. Protein abundance ratios were measured with iTRAQ, and proteins with fold change ratios ≥1.5 or ≤0.667 were considered differentially expressed. Finally, the Gene Ontology (GO) analysis and Kyoto Encyclopedia of Genes and Genomes (KEGG) analysis were performed for the differential expression of proteins, of which the GO analysis was performed by the GOseq R package, and GO terms *p* < 0.05 were considered significantly enriched by differentially expressed proteins. The statistical enrichment of the differential expression of proteins in KEGG pathways was tested using the KOBAS software (Beijing BangFei Bioscience Co., Ltd., Beijing, China).

## 3. Results

### 3.1. Amino Acid Abundance

Seventeen amino acids were detected in tenderloin and flank steaks in this study, including seven essential amino acids (Thr, Val, Met, Ile, Leu, Phe and His) and 10 non-essential amino acids (Tyr, Asp, Lys, Arg, Pro, Ser, Glu, Gly, Ala and Cys). The abundance of Gly, Cys, Ile, Lys, and Pro varied most between tenderloin and flank steaks (*p* < 0.05; Table 1).

### 3.2. Identification and Differential Expression of Proteins

**Figure 1:**
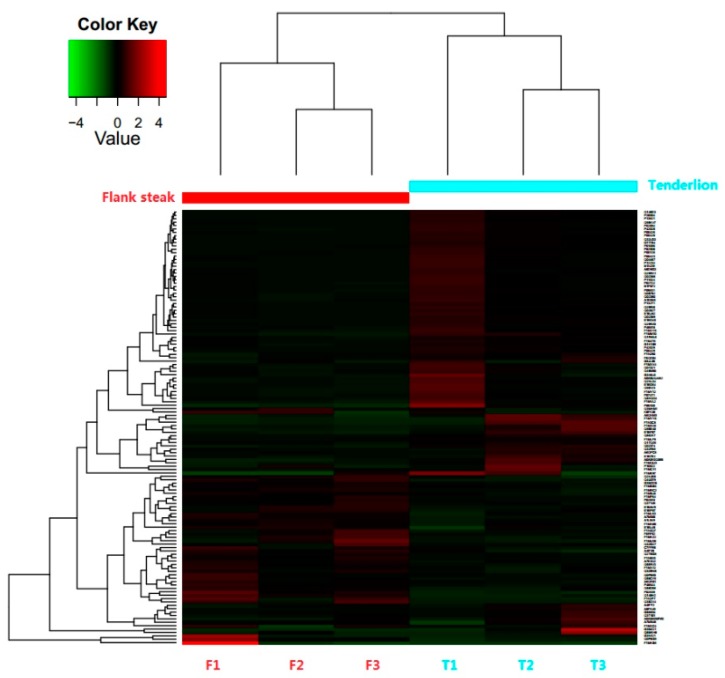
Hierarchical clustering of differentially expressed proteins (DEPs) between tenderloin and flank steaks in beef cattle. Each row represents one protein. Columns F1, F2, and F3 represent samples from three technical replicates for flank steaks, and columns T1, T2, and T3 represent samples from three technical replicates for tenderloin steaks, and flank steaks in beef cattle samples belong to group F, whilst tenderloin steaks in beef cattle samples belong to group T. The expression levels of proteins are shown in red (up-regulated) and green (down-regulated).

A total of 15,255 distinct peptides and 1663 proteins were detected after filtering with a false discovery rate (FDR) ≤0.01. In addition, 128 differentially expressed proteins (DEPs) were identified (based on log2 fold change ≥1.5 or log2 fold change ≤0.667), of which 44 were up-regulated and 84 were down-regulated (Supplementary Table S1). The results of a hierarchical clustering analysis of the 128 DEPs showed that the three biological duplicate samples clustered into a single group (Figure 1). Furthermore, 27 prominent proteins were selected from the 128 DEPs (*p* < 0.05) for bioinformatics analysis (Table 2), of which 101 proteins show differential expression patterns between the two steaks, however, according to the statistical analysis, these differences are not statistically significant (*p* > 0.05).

**Figure 2:**
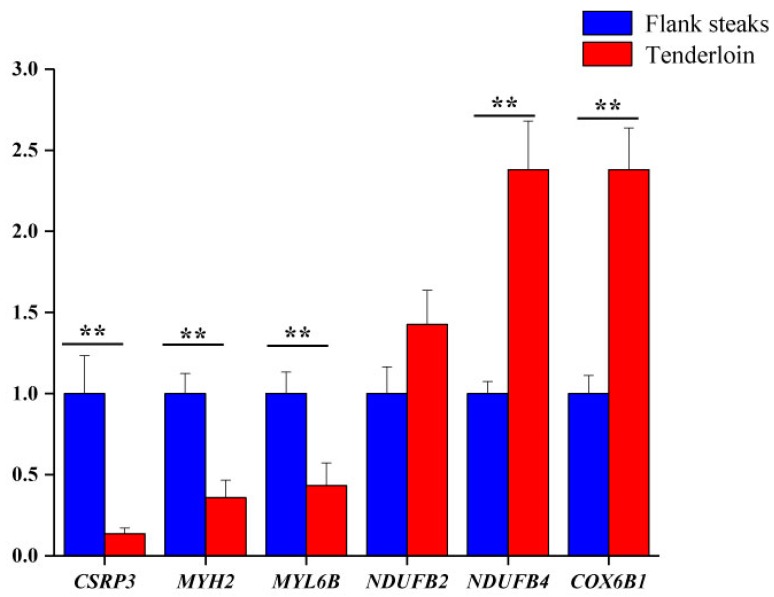
Validation of the gene corresponding to the differentially expressed proteins by quantitative real-time reverse transcription PCR (qRT-PCR). ** very significant differences between the tenderloin steaks and the flank steaks (*p* < 0.01).

**Figure 3:**
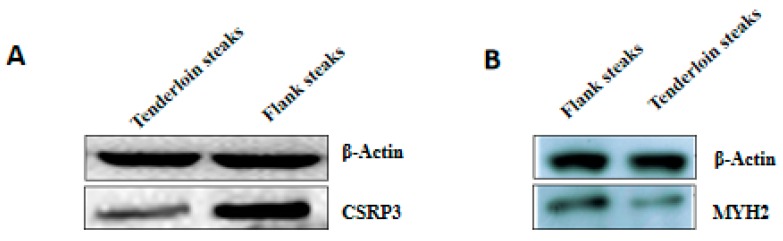
The relative expression levels of CSRP3 (**A**) and MYH2 (**B**) proteins between tenderloin and flank steaks in beef cattle. β-actin was used as an internal reference protein.

Six genes corresponding to the differentially expressed proteins were selected for qRT-PCR analysis to validate their differential expression. The expression levels of *CSRP3*, *MYH2*, and *MYL6B* genes were lower in tenderloin steaks than flank steaks, whereas the expression of *NDUFB2*, *NDUFB4*, and *COX6B1* genes were higher in the tenderloin steaks (Figure 2). The expression levels of five of the selected genes (*CSRP3*, *MYH2*, *MYL6B*, *NDUFB4*, and *COX6B1*) were significantly different between the tenderloin steaks and flank steaks, and the trend of expression levels of *NDUFB2* gene was consistent with the iTRAQ results. Western Blot analysis indicated that the expression of CSRP3 and MYH2 proteins was higher in the flank steaks (Figure 3).

### 3.3. Functional Annotation of DEPs

**Figure 4:**
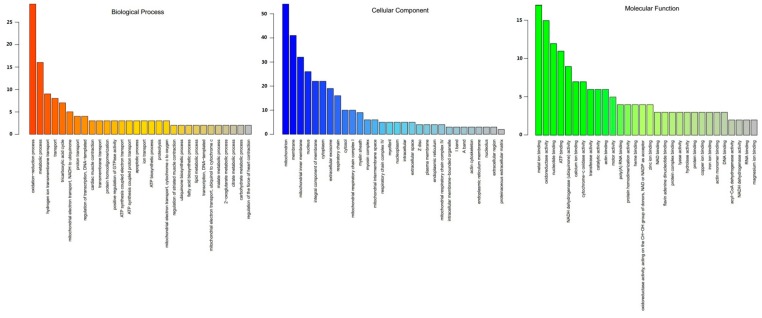
Gene ontology (GO) distribution of DEPs between tenderloin and flank steaks. DEPs are grouped into 30 categories according to biological process, cellular component and molecular function. The X-axis is the GO term, and the Y-axis is the number of differentially expressed proteins.

**Figure 5:**
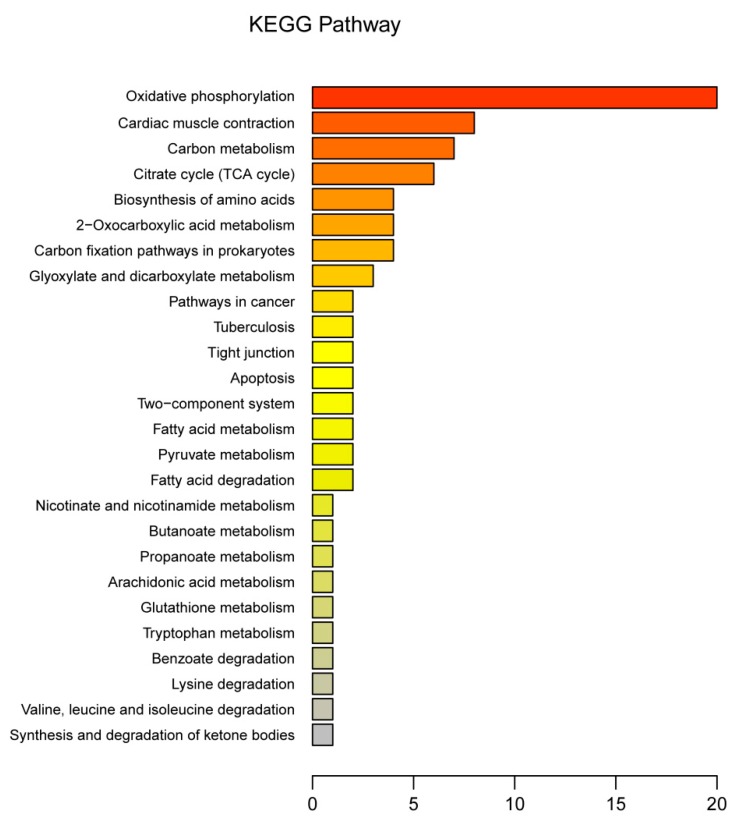
Kyoto Encyclopedia of Genes and Genomes (KEGG) pathway analysis of protein. The X-axis is the number of differentially expressed proteins enriched on this pathway.

The 128 DEPs between tenderloin and flank steaks were functionally grouped into 30 annotation clusters. GO annotation of biological processes showed that most of the DEPs were linked to oxidation-reduction, metabolism, hydrogen ion transmembrane transport, transport, the tricarboxylic acid (TCA) cycle, mitochondrial electron transport, and the conversion of NADH to ubiquinone. The GO terms associated with the cellular component category indicated that many DEPs were related to mitochondria, membrane, mitochondrial inner membrane, and nucleus subcategories. For molecular function, metal ion binding, oxidoreductase activity, nucleotide binding and ATP binding were the main enriched GO terms (Figure 4). The KEGG pathway enrichment analysis indicated that 128 DEPs were related to oxidative phosphorylation, cardiac muscle contraction, carbon metabolism, the TCA cycle, the biosynthesis of amino acids, 2-oxocarboxylic acid metabolism, carbon fixation pathways in prokaryotes, glyoxylate and dicarboxylate metabolism, tight junctions, apoptosis, two-component systems, fatty acid metabolism, pyruvate metabolism, fatty acid degradation, nicotinate and nicotinamide metabolism, butanoate metabolism, propanoate metabolism, arachidonic acid metabolism, glutathione metabolism, tryptophan metabolism, lysine degradation, and valine, leucine, and isoleucine degradation (Figure 5). Thus, the DEPs were mainly associated with the energy and amino acid metabolism.

## 4. Discussion

Muscles are of critical importance to energy, protein, and lipid metabolism. The content, composition and active functions of muscle components have significant effects on the formation and quality of meat. Closely related to life activities, amino acids are the basic components of proteins and are indispensable nutrients in all organisms. Branched chain amino acids (BCAA), including leucine, isoleucine, and valine, are essential amino acids that are mainly oxidised in muscle [14]. These BCAAs take part in the TCA cycle through ketogenic and glycaemic effects, ensuring the conversion of the three major nutrients (proteins, carbohydrates, and lipids) in the body. Numerous studies have shown that BCAAs play a vital role in energy balance and lipid metabolism [15,16], and BCAA oxidation is an important source of energy in animals. However, most studies have focused on leucine. In the present study, the content of isoleucine in flank steaks was significantly higher than that in tenderloin steaks (*p* < 0.05), which suggests that isoleucine may serve as an energy regulating substance that modulates the energy metabolism, thereby affecting lipid deposition between different muscle types. In addition, the content of cysteine and glycine was greater in flank steaks than tenderloin steaks.

An analysis of significant GO categories indicated that most DEPs are likely to be correlated with the energy metabolism in mitochondria. Mitochondrial oxidative phosphorylation, carried out by the respiratory chain in the mitochondrial inner membrane, is an important source of energy in all organisms. The mitochondrial respiratory chain is composed of three catalytic complexes, NADH:ubiquinone (Q) oxidoreductase (Complex I), ubiquinol:ferricytochrome c oxidoreductase (Complex III), and ferrocytochrome c:O2 oxidoreductase (Complex IV), plus two lower molecular weight redox carriers cytochrome c and ubiquinone-10 (Q) [17]. In the present study, we detected a decrease in the expression of NADH dehydrogenase [ubiquinone] 1 beta subcomplex subunit 2 (NDUFB2), NADH dehydrogenase [ubiquinone] 1 beta subcomplex subunit 4 (NDUFB4), isocitrate dehydrogenase [NAD] subunit beta, and mitochondrial (IDH3B) and cytochrome c oxidase subunit 6B1 (COX6B1), all of which are related to oxidation-reduction processes. These results indicate that the oxidation respiratory chain may be weaker in flank steaks than tenderloin steaks.

The composition and type of myofibers is an important source of variation in meat quality [18]. Muscle myosin is a large molecule made of six polypeptide chains, consisting of two identical heavy chains and two pairs of light chains [19]. Based on differences in the sensitivity of acto-myosin ATPase activity to pH preincubation, four main fiber types have been identified by histochemistry in adult skeletal muscle: I, IIA, IIX (or IID), and IIB [20]. Four MyHC isoforms have been identified in skeletal muscle, including type I (slow-twitch, red muscle, oxidative), IIa (fast-twitch, red muscle, oxidative), and IIx/IIb (fast-twitch, white muscle, glycolytic) [21,22]. The speed of contraction increases in the rank order I <IIa <IIx <IIb [23]. Previous studies revealed variation in the type of fibers and myosin isoforms present in Longissimus dorsi and Semitendinosus muscles in buffalo [24]. In the present study, myosin heavy chain 2 (MYH2 or MyHC IIa) was up-regulated in flank steaks compared with tenderloin steaks in Simmental cattle. This difference in isoforms may be related to the functions of different muscles. Flank steaks are made from respiratory pump muscle that is used constantly, while tenderloin muscle is used more infrequently. Myosin light chain 6B (MYL6B) is involved in myofibril and muscle myosin development. Xu et al. (2014) found that MYL6B is mainly enriched in myofibrils, muscle myosin complexes, and structural constituents of muscle based on the co-expression analysis of foetal weight-related genes in ovine skeletal muscle during mid and late foetal developmental stages [25]. In the present work, myosin light chain 6B (MYL6B) was up-regulated in flank steaks compared to tenderloin steaks in Simmental cattle. Thus, MYL6B may help to explain the differences in meat quality between flank and tenderloin steaks.

Cysteine and glycine-rich protein 3 (CSRP3), a member of the cysteine-rich protein family, is highly expressed in striated muscle [26,27], and is critical for muscle proliferation and differentiation [28]. This protein was up-regulated in flank steaks compared to tenderloin steaks. Interestingly, combined with the results of the amino acid analysis described above, this allows us to conclude that the abundance of cysteine and glycine is greater in flank steaks.

Two core metabolic pathways, glycolysis and oxidative phosphorylation, are involved in ATP production for energy, and glucose, fatty acids, and amino acids are the main energy substrates involved in mitochondrial oxidation [29]. In the present study, 20 DEPs related to oxidative phosphorylation were downregulated in tenderloin steaks, indicating a high level of energy metabolism in this muscle.

## 5. Conclusions

In summary, we identified 17 amino acids and 128 DEPs in bovine flanks and tenderloin steaks, and Gly, Cys, Ile, Lys, and Pro differed most greatly between the meat types (*p* < 0.05). Importantly, many DEPs are related to energy and amino acid metabolism. Thus, our findings provide a useful resource for further investigating the roles of DEPs and amino acid abundance in determining the characteristics and quality of meat from cattle. Such future studies will enable a better understanding of the molecular mechanisms underpinning amino acid composition in beef.

## Figures and Tables

**Table 1:** Differences in amino acid abundance between flank and tenderloin steaks from Simmental cattle.

Items	Flank	Tenderloin	*p*-Value
Gly	4.73 ± 0.15	3.30 ± 0.15	0.002
Cys	1.00 ± 0.06	0.73 ± 0.03	0.016
Ile	4.93 ± 0.15	3.47 ± 0.15	0.002
Lys	6.90 ± 0.15	5.63 ± 0.23	0.010
Pro	3.70 ± 0.10	2.57 ± 0.15	0.003

**Table 2:** Proteins up- and down-regulated between group T (tenderloin steaks from beef cattle) and group F (flank steaks from beef cattle).

Gene Name	Protein Name	Fold Change	*p*-Value
		Group F/Group T	
*S100A1*	Protein S100-A1	3.92	0.036
*MYL6B*	Myosin, light chain 6B, alkali, smooth muscle and non-muscle	3.31	0.012
*CSRP3*	Cysteine and glycine-rich protein 3	2.70	0.006
*SCHIP1*	Schwannomin-interacting protein 1	2.57	0.021
*DPYSL3*	DPYSL3 protein	1.93	0.004
*MYH2*	Myosin-2	1.92	0.006
*COG6*	Conserved oligomeric Golgi complex subunit 6	1.88	0.015
*MVP*	Major vault protein	1.76	0.005
*HSPA2*	Heat shock 70kDa protein 1A	1.65	0.043
*CRYAB*	Alpha-crystallin B chain	1.61	0.039
*CCDC178*	Uncharacterised protein (Fragment)	1.55	0.010
*UCHL3*	Ubiquitin carboxyl-terminal hydrolase isozyme L3	1.54	0.047
*HSPB7*	Uncharacterised protein (Fragment)	1.53	0.042
*SENP5*	Uncharacterised protein	1.51	0.026
*SDPR*	SDPR protein (Fragment)	0.66	0.016
*TRNT1*	Uncharacterised protein	0.65	0.033
*UBE2E3*	Ubiquitin-conjugating enzyme E2 E3	0.64	0.003
*CKMT2*	Creatine kinase S-type, mitochondrial	0.64	0.034
*NDUFA8*	NADH dehydrogenase [ubiquinone] 1 alpha subcomplex subunit 8	0.64	0.037
*NDUFB4*	NADH dehydrogenase [ubiquinone] 1 beta subcomplex subunit 4	0.64	0.021
*IDH3B*	Isocitrate dehydrogenase [NAD] subunit beta, mitochondrial	0.63	0.041
*COX6B1*	Cytochrome c oxidase subunit 6B1	0.62	0.023
*COX17*	Uncharacterised protein	0.61	0.037
*PCDHGB5*	Uncharacterised protein	0.58	0.030
*CYP1A1*	Cytochrome P450 (Fragment)	0.42	0.016
*NDUFB2*	NADH dehydrogenase [ubiquinone] 1 beta subcomplex subunit 2	0.40	0.002
*ARRDC4*	Arrestin domain-containing protein 4	0.30	0.042

## Supplementary Materials

The following are available online at https://www.mdpi.com/2076-2615/10/1/150/s1, Figure S1: QPCR Primers used in the validation of the genes corresponding to the differentially expressed proteins.
