# Supplementary material for: Proteomic Analysis of Beef Tenderloin and Flank Assessed Using an Isobaric Tag for Relative and Absolute Quantitation (iTRAQ)
Source: Animals (Basel). 2020 Jan 16;10(1):150. doi: 10.3390/ani10010150 (PMC7022852; doi:10.3390/ani10010150)
Supplement: Supplementary file 1 [file animals-10-00150-s001.pdf]

Table S1. QPCR Primers used in the validation of the genes corresponding to the differentially expressed proteins.

Gene	Sense primer seq 5'–3'	Anti-Sense primer seq 5'–3'	Production lenth	Temperature
NDUFB2	CTTTGTCTCACTGTGGGTCG	CATTTTGGAGTTTCCGCCTGA	309bp	56 °C
NDUFB4	GACCCAGCTGAATACGACA	CTGATCTTGCGTAGGTCCAA	166bp	55 °C
MYH2	GATCTCTGACCTCACGGAGCA	CTGCATGGACTCCACGACT	271bp	59 °C
MYL6B	TCGAGTTTAACAAGGACCAGC	TCTTTGTCAAACACCCGAAGC	292bp	59 °C
CSRP3	CTGAGCCGACACAGATCACA	CTTGTCCATACCCGATCCCT	281bp	57 °C
COX6B1	ACTATGGCAGAAGACATCCAG	GTACCATTCACACACGGAGA	174bp	55 °C
